# Maternal Inflammation with Elevated Kynurenine Metabolites Is Related to the Risk of Abnormal Brain Development and Behavioral Changes in Autism Spectrum Disorder

**DOI:** 10.3390/cells12071087

**Published:** 2023-04-04

**Authors:** Yuki Murakami, Yukio Imamura, Yoshiyuki Kasahara, Chihiro Yoshida, Yuta Momono, Ke Fang, Daisuke Sakai, Yukuo Konishi, Toshimasa Nishiyama

**Affiliations:** 1 Department of Hygiene and Public Health, Kansai Medical University, Hirakata 573-1010, Japan; 2 Department of Architecture and Architectual Systems Engineering, Graduate School of Engineering, Kyoto University, Kyoto 615-8530, Japan; 3 Department of Traumatology and Acute Critical Medicine, Graduate School of Medicine/Faculty of Medicine, Osaka University, Suita 565-0871, Japan; 4 Department of Maternal and Fetal Therapeutics, Tohoku University Graduate School of Medicine, Sendai 980-8575, Japan; 5 Department of Biology, Kanazawa Medical University, Kanazawa 920-0293, Japan; 6 Center for Baby Science, Doshisha University, Kyotanabe 619-0225, Japan; 7 Healthcare and Medical Data Multi-Level Integration Platform Group, RIKEN Medical Sciences Innovation Hub Program, Yokohama 230-0045, Japan

**Keywords:** interleukin-17a, maternal immune activation, autism spectrum disorder, kynurenine

## Abstract

Several studies show that genetic and environmental factors contribute to the onset and progression of neurodevelopmental disorders. Maternal immune activation (MIA) during gestation is considered one of the major environmental factors driving this process. The kynurenine pathway (KP) is a major route of the essential amino acid L-tryptophan (Trp) catabolism in mammalian cells. Activation of the KP following neuro-inflammation can generate various endogenous neuroactive metabolites that may impact brain functions and behaviors. Additionally, neurotoxic metabolites and excitotoxicity cause long-term changes in the trophic support, glutamatergic system, and synaptic function following KP activation. Therefore, investigating the role of KP metabolites during neurodevelopment will likely promote further understanding of additional pathophysiology of neurodevelopmental disorders, including autism spectrum disorder (ASD). In this review, we describe the changes in KP metabolism in the brain during pregnancy and represent how maternal inflammation and genetic factors influence the KP during development. We overview the patients with ASD clinical data and animal models designed to verify the role of perinatal KP elevation in long-lasting biochemical, neuropathological, and behavioral deficits later in life. Our review will help shed light on new therapeutic strategies and interventions targeting the KP for neurodevelopmental disorders.

## 1. Introduction

Maternal inflammation with elevated kynurenine (Kyn) metabolites has been implicated in developing various central nervous system (CNS) disorders. Kyn metabolites are produced as part of the tryptophan (Trp) catabolism pathway, and increased levels of these metabolites have been found in response to inflammation. Maternal inflammation during pregnancy can increase in pro-inflammatory cytokines, which can stimulate the production of Kyn metabolites.

Studies have shown that elevated inflammation and Kyn metabolites during pregnancy are associated with an increased risk of several CNS disorders, including autism spectrum disorder (ASD) [1,2,3], schizophrenia (SCZ) [4,5], and depression [3,6,7]. In ASD patients, for example, alterations in the Kyn pathway (KP) have been observed in both the peripheral and central nervous systems [1,2,8]. It has been shown that inflammation during pregnancy may contribute to the dysregulation of the KP and increase the risk of ASD. Similarly, elevated levels of Kyn metabolites, especially kynurenic acid (Kyna), have been observed in individuals with SCZ [9,10,11]. It has been shown that inflammation and oxidative stress may contribute to the dysregulation of the KP in this disorder. In depression, altered levels of Kyn metabolites have also been reported, with higher levels of Kyn and lower levels of its downstream metabolite, Kyna, observed in individuals with depression [12,13]. The relationship between maternal inflammation with elevated Kyn metabolites and CNS disorders is complex and poorly understood. However, studies show that dysregulation of the KP because of inflammation may contribute to the development of various CNS disorders.

ASD is a complex developmental disorder associated with impaired social interactions/affective function, communication abnormalities, stereotypic behavior, a repetitive repertoire of interests and activities [14], and secondary causes of cognitive impairment and depression. In 2018, the Autism and Developmental Disabilities Monitoring (ADDM) Network reported that one in 44 (23.0 per 1000) children aged 8 years were estimated to have ASD, and the ASD prevalence has kept increasing in the last decade [15,16]. Additionally, several genetic factors of ASD have been identified, and twin studies estimate its heritability to be 64–91% [17,18]. However, maternal immune activation (MIA) following viral infection is associated with an increased ASD prevalence and is one of the most impacting environmental risk factors [19,20,21].

Recent studies have demonstrated that the neuroimmune response and the abnormality of KP metabolites are linked to the pathophysiology of ASD. Some evidence demonstrates that children with ASD show increased levels of serum inflammatory cytokines [8,22]. As the KP of Trp degradation is activated in neuroinflammatory states, previous studies also demonstrate inflammation-induced KP activation in children with ASD [1,8]. Activation of the KP can generate various endogenous neuroactive KP metabolites, such as Kyn and Kyna. In particular, increasing evidence shows that these metabolites play important and unique roles in brain development [23]. We summarize the possible relationship between the KP, as well as its neuroactive metabolites, and inflammation and also the risk of abnormal brain development and behavioral changes to provide insights into the pathological mechanisms of ASD.

## 2. Interleukin (IL)-17a as a Potential Mediator of ASD

### 2.1. Pro-Inflammatory Cytokines and ASD

An altered and abnormal immune system has been widely observed in the periphery of patients with ASD and in experimental animal models. Several studies have found chronic neuroinflammation to be associated with ASD, indicated by an increased number of activated microglia and astrocytes, and the production of cytokines and chemokines in the brain [8,24,25,26,27,28]. Additionally, ASD has been associated with chronic inflammatory and allergic conditions, such as maternal infection and autoimmune diseases in the first trimester of pregnancy [19,29,30,31]. In the brain of the patient with ASD, IL-6 elevation was a repeated finding [24,27,32], and consistent with these findings, Smith et al. showed that maternal IL-6 was critical for mediating the behavioral and transcriptional changes in the offspring using IL-6 blocking antibody and IL-6 gene-deficient mice [33]. Moreover, Rudolph et al. indicated that functional connectivity within and between multiple neonatal brain networks could be modeled to estimate the maternal IL-6 concentration during pregnancy [34]. However, IL-6 is a multifaceted signal capable of triggering the expression of other cytokines and immune regulatory genes that may additionally impact the developing brain or are responsible for precipitating ASD. T helper (Th)17 cells have been proposed to play important roles in immune responses against extracellular bacteria and fungi. Their dysregulation is related to various autoimmune diseases, such as allergies, asthma, rheumatoid arthritis, and inflammatory bowel diseases (IBDs) [35,36]. The differentiation of Th17 cells from naïve Th cells is initiated by stimulation with professional antigen-presenting cells (APCs) and particular cytokines, including IL-6, IL-21, and transforming growth factor-beta (TGF-β) [36,37,38,39]. Maturated Th17 cells produce the signature cytokines IL-17a, IL-17f, and IL-21, essential for mucosal host defense against extracellular bacteria and fungi, and recruit neutrophils by inducing chemokines under inflammation [36]. Recently, cytokines produced by Th17 cells have been shown to have a role in ASD. IL-17a is the predominant Th17 cytokine, and elevated levels of IL-17a have been found in the blood and correlated with the severity of behavioral symptoms in individuals with ASD [8,28,40,41]. A MIA model mouse injected intraperitoneally with synthetic double-stranded RNA [polyinosinic:polycytidylic acid; poly(I:C)], a mimic of viral infection, showed increased levels of IL-17a in the maternal blood and postnatal fetal brain [42,43]. Offspring from poly(I:C)-treated dam showed ASD-like behaviors, such as abnormal communication and social interest, and increased stereotypic and anxiety-like behaviors [44]. Inhibited IL-17a signaling by antibody blockade of the cytokine in poly(I:C)-treated pregnant mice can prevent ASD-like phonotypes in the offspring [42].

Additionally, mice directly injected with IL-17a into the fetal lateral ventricles on embryonic day (E) 14.5 showed similar ASD-like behaviors and cortical disorganization as the offspring of poly(I:C)-treated dam [42], and activated cortical microglia, which excessively phagocytosed neuronal progenitor cells in the ventricular zone [45]. Furthermore, chronic gestational IL-17a causes ASD-like phenotypes early and persistently in male offspring and leads to 320 differentially expressed genes related to “neuron-neuron synaptic transmission” and “cell cycles” [46]. IL-17a also blocks the proliferation of neuronal stem cells, resulting in a significantly reduced number of astrocytes and oligodendrocyte precursor cells [47]. Kim et al. showed that ASD phenotypes following MIA in offspring necessitate maternal intestinal bacteria, including segmented filamentous bacteria (SFB), to promote Th17 cell differentiation and produce IL-17a [48]. Pretreatment of antibiotic vancomycin in poly(I:C)-injected dam canceled the development of all behavioral abnormalities in MIA-offspring. Generally, in all the findings, dysregulation of maternal Th17 differentiation or the Th17/IL-17a pathway may play a critical role in the pathophysiology of MIA-induced ASD.

### 2.2. Proinflammatory Cytokines Regulate KP Enzymes

The correlation between proinflammatory cytokines and KP enzymes is well-known. Indoleamine 2,3-dioxygenase1 (IDO1) is an enzyme that is the first rate-limiting step of Trp degradation and leads to the production of a series of downstream metabolites in KP. During pregnancy, IDO1 is mainly expressed in the trophoblast [49] and placenta [50] to protect the fetus from the attack of the maternal immune system by inhibition of T-lymphocyte responses through Trp consumption and Trp catabolism defects [51,52]. In maternal circulation, the ratio of Kyn and Trp (Kyn/Trp ratio) is also significantly increased in a normal pregnancy at late gestation stages, compared to non-pregnant women [53]. CD4-positive Th cells may release various cytokines after stimulation, such as classified Th1-, Th2-, and Th17-type cytokines. The predominant Th2 immunity, which overrules the Th1 immunity at the placental implantation site, protects the fetus by balancing the Th1 immunity and accommodates fetal and placental development [54]. IL-6 is a major Th2-type cytokine, which can induce the upregulation of IDO1 in the chorionic villi and decidua of women in early pregnancy, revealing that Th-2type cytokines can induce maternal immunotolerance via activated IDO1 [55,56]. In the normal physiological state of very early gestation (around 8 weeks of gestation), IL-6 upregulates the IDO1 expression in chorionic villi and decidua by enhancing SC-43-activated SH2-domain-containing phosphatase (SHP)-1/2 expression via signal transducers and activators of transcription (STAT) 3 and phosphorylated-STAT3 [57], and high expression of IL-6 may help promote immunological tolerance and a successful pregnancy. However, Th2 cells may participate in autoantibody production and enhance autoimmunity. In addition, overly increased tolerogenic signals from Th2 cells may induce uncontrolled viral infections [54]. The fetal ZIKA virus infection causes microcephaly, and an association is reported with ASD, along with the discovery of the predominant expression of Th2 cytokines, including IL-6, in the meninges, perivascular space, and parenchyma [54,58]. These findings show that adequate timing and properly immuno-activated Th2 are important for immunotolerance and fetus protection from infection.

Tryptophan 2,3-dioxygenase (TDO) is the other key enzyme in the KP, and it is mainly expressed in the liver and maintains systemic Trp levels by degrading excess dietary Trp under normal physiological conditions [59,60]. TDO is expressed pathologically in various tumors [61]. Kyn, catabolized from Trp by IDO1 and TDO, suppresses antitumor immune responses and promotes tumor-cell survival [62]. TDO was also identified in pericytes and interstitial syncytiotrophoblasts in the human placenta [63], and continuous TDO expression was observed in mouse decidual stromal cells, starting at E3.5 until gestation end. Despite the highly expressed TDO in decidual stromal cells, TDO-deficient and IDO1/TDO-double-deficient female mice did not show increased rates of miscarriage because of an absence of an increased immune attack against allogenic fetuses [60]. These results show that TDO may not be a dominant mechanism of maternal immunotolerance able to compensate for the absence of IDO1. IL-1β is a multifunctional cytokine and one of the first cytokines released by macrophages, monocytes, and dendritic cells during an infection. In addition, IL-1β is essential for efficient innate and adaptive immune responses [64], and previous research shows that IL-1β stimulates KP and regulates the production of IL-6 secretion by increasing the TDO expression [65]. However, little information is available on regulating IL-1β and TDO in normal pregnancy. A few reports on tumor immunology indicate a relationship between inflammation and TDO induction, and TDO has immunomodulatory functions in promoting tumor resistance and proliferation [61,62,65,66,67].

In the peripheral blood of healthy pregnant women, Th17 cells are rare among CD4-positive T cells (0.64–1.4%), and the number of circulating Th17 cells does not change during pregnancy [68]. Another study reported that pregnant women in the third trimester presented with a decreased proportion of Th17 cells compared to non-pregnant women [69]. The decidua contains a higher density of Th17 cells than the peripheral blood [68]. The decidual IL-17a-positive cell count was consistent with the neutrophil count, showing that IL-17a-positive cells are intimately involved in neutrophil infiltration [70] and induce protective immunity against extracellular microbes in the uterus. The paradoxical association between IL-17a and the induction of IDO1 in opportunistic infections is well known. In a fungal infection with physiological conditions, the IL-17a pathway downregulated the Trp catabolism and completely antagonized the induction IDO1 by interferon-gamma (IFN-γ) in neutrophils [71]. Although little information is available on the regulation of IL-17a and KP in normal pregnancy, Krause et al. demonstrated that candidemic patients with antibiotic therapy had significantly higher IL-17a and Kyn levels than non-candidemic patients [72]. In addition, we demonstrated that maternal overexpression of IL-17a induced significantly elevated levels of Kyn and KP metabolites in maternal serum and fetal plasma [3].

Cytokines and KP metabolites are closely associated with mediating CNS and immune system communication. Both molecules regulate not only neuronal cells, such as neurons and in glia activity, but also several immune cells, such as in leukocytes activity. Stone et al. show that cytokines and KP metabolites perform complementary functions, generating an integrated network related to some neuroimmune communications [73]. The KP is a key to understanding how systemic inflammation can affect brain function. Additionally, the KP metabolites can influence the surveillance, defensive, and tolerance activities of the immune system. Overall, KP and its metabolites may play key roles in the pathogenesis of normal and abnormal pregnancies. The induction of KP by inflammation may influence the neurodevelopment of the fetus.

## 3. The Metabolism of Trp in the Intestine and the Role of Microbiota in ASD

### 3.1. Intestinal Trp Metabolism Pathways and Physiological Roles

Trp metabolism follows three major pathways in the gastrointestinal (GI) tract: (1) the KP in both host immune cells and epithelial cells via IDO1 (details in next part) [74]; (2) the serotonin (5-hydroxytryptamine; 5-HT) production pathway in host enterochromaffin cells via Trp hydroxylase (THP) 1 [75]; and the direct transformation of Trp into several molecules, including aryl hydrocarbon receptor (AhR) ligands, by the gut microbiota [76,77,78].

TPH2 produces the neurotransmitter 5-HT, which plays an important role in the brain (see the other part). More than 90% of the body’s 5-HT is produced in the intestine through TPH1. Peripheral 5-HT triggers various functions in the GI tract and is implicated in a wide range of physiological functions through its activation of specific 5-HT receptors [79,80]. Intestinal 5-HT has been found to modulate intrinsic or extrinsic neurons and to influence intestinal peristalsis and the motility, secretion, vasodilation, and absorption of nutrients [79,81,82]. Additionally, intestinal 5-HT was shown to have a hormonal role in bone formation by studies of the low-density receptor-related protein (LRP) 5, which works with its co-receptors to activate the Wnt-β-catenin signaling pathway [83]. The gut microbiota also produces major intestinal 5-HT [75]. Germ-free mice exhibit significantly decreased colonic and fecal 5-HT production levels and low blood concentration of 5-HT [75,84]. The mechanisms of how the gut microbiota regulates 5-HT production remain unknown. However, a few studies demonstrated that some metabolites, such as short-chain fatty acids (SCFAs) or secondary bile acids, can modulate 5-HT biosynthesis [75,85].

The degradation of dietary proteins leads to the release of Trp, which is directly converted by gut microorganisms into various metabolites, such as indole and its derivatives. Many indole derivatives, including indole acrylic acid (IA), indole-3-acetaaldehyde (IAAld), indole-3-acetic acid (IAA), indole-3-propionic acid (IPA), and indole-3-aldehyde (IAld), are ligands for AhR [86,87]. Only a few commensal species can produce AhR ligands, such as *Lactobacillus spp* and *Peptostreptococcus russellii* [76,88,89]. AhR is a key receptor, which regulates the immune response at barrier sites, barrier integrity, and homeostasis by acting on epithelial renewal and many immune cells’ differentiation [90]. Especially, Trp metabolites play an important role in the differentiation and function of several immune cells, such as T-regulatory cells (Tregs), B-regulatory cells (Bregs), IL-22-producing innate lymphocyte cells 3 (ILC3s), and anti-inflammatory macrophages in the gut, because Th17 cells, Tregs, B cells, and APCs express AhR [91]. Trp metabolizing pathways have been identified in other species, such as *Clostridium sporogenes*, *Clostridium limosum*, *Escherichia coli* (*E. coli*), *Enterococcus faecalis*, and *Bacteroides ovatus* [78,89,92]. *Clostridium sporogenes* can decarboxylate Trp to produce the neurotransmitter tryptamine [92]. In the brain, tryptamine has been shown to activate trace dopaminergic, serotonergic, and glutamatergic systems [93]. In the GI tract, tryptamine activates the 5-HT_4_ receptor and regulates GI motility [94]. *Clostridium sporogenes* also oxidate and reduce to produce IAA and IPA, which are known to affect intestinal permeability and host immunity [90,95,96]. *Clostridium limosum*, *E. coli*, *Enterococcus faecalis*, and *Bacteroides ovatus* can convert Trp into indole [78,97], an interspecies signaling molecule that can control aspects of bacterial physiology, such as sporulation, antibiotic resistance, and biofilm formation.

### 3.2. The Regulation of Trp Metabolism by Gut Microbiota

Gut microbiota can influence the KP via the alteration of Trp availability or the regulation of the immune system, which can affect the activity of IDO1. In germ-free mice or mice with an altered microbial composition due to antibiotics, the plasma levels of Trp were increased, while the levels of KP metabolites and the peripheral 5-HT levels were decreased. Consistent with these changes, the Kyn/Trp ratio was reduced, which indicated the lower activity of IDO1 and TDO. Notably, the induction of some gut microbiotas, such as *Bifidobacterium infantis*, could restore the normal activity of these two enzymes [74,75,98,99,100,101]. The gut microorganism can degrade Trp into several metabolites that consequently limit the availability of Trp for the KP and 5-HT pathways, as shown in the previous paragraph. 

Gut microbiota and IDO1 also have feedback control over each other. IDO1 can induce an immunosuppressive response in the GI tract by regulating immune reactivity and microbial metabolism. Contrarily, gut microbiota can change the amount of KP metabolites and IDO1 activity by limiting Trp usage. Additionally, some metabolites derived from microbiota have anti-inflammatory effects and modulate the immune system and KP via the regulation of IDO1. SCFAs are one of the major metabolites from microbiota and play a significant role in intestinal homeostasis. It has been demonstrated that butyrate (one of the SCFAs) can downregulate the expression of STAT1, which is one of the main mediators of IDO1 expression [102]. Decreased STAT1 expression inhibits IFN-γ-dependent STAT1 phosphorylation and subsequently reduces the STAT-1-dependent transcriptional activity of IDO1. SCFAs also can inhibit histone deacetylase (HDAC) [103], and the downregulation of HDAC could suppress the production of several proinflammatory cytokines, such as tumor necrosis factor (TNF)-α, IFN-γ, and IL-6 [104,105]. Therefore, SCFAs could inhibit IDO1 activity in an indirect manner.

### 3.3. Dysbiosis in ASD

Among several comorbidities in ASD, GI distress is reportedly related to the prevalence of and a correlation with symptom severity. Dysbiosis of the microbiota is implicated not only in the pathogenesis of ASD, but also in several chronic diseases, such as IBD, allergy, asthma, cardiovascular disease, obesity, and diabetes mellitus. Especially, evidence of microbial dysbiosis in ASD has been growing in the last two decades [106,107,108,109,110,111,112,113,114,115]. A recent systematic review shows that ASD patients had an elevated abundance *Proteobacteria*, *Clostridium*, and *Bacteroides*, while they had lower levels of *Bifidobacterium*, *Prevotella*, and *Blautia*, compared to healthy controls [116]. The elevated abundance of *Proteobacteria* is associated with host inflammation because this microbe can produce lipopolysaccharide (LPS). *Clostridium* also can produce pro-inflammatory toxins and propionic acid that may be related to the severity of ASD symptoms [117,118]. Sandler et al. showed that oral vancomycin treatment improved behavioral and GI symptoms in ASD children by reducing *Clostridium* [106]. *Bacteroides* produce SCFAs, especially propionic acid, and SCFAs are involved in the proper function of the gut immune system through the modulation of gene expression. Therefore, an imbalance in the concentration of SCFAs can alter the gut homeostasis and trigger peripheral inflammation. SCFAs can cross the blood–brain barrier (BBB) via monocarboxylate transporters located on endothelial cells and influence brain development by modulation of 5-HT and dopamine production [119,120]. Contrarily, some *Bifidobacterium* species synthesize gamma-amino butyric acid (GABA), which is found in lower concentrations in ASD children. *Prevotella* species have essential genes for the biosynthesis of vitamin B1, which was reported to palliate ASD symptoms [121,122]. *Blautia* species have roles in bile acid and Trp metabolism in the intestine, which is related to 5-HT synthesis and accelerating GI motility [75,123].

In addition to immune and GI dysfunction that may be linked to dysbiosis, there is some evidence that altering the microbiota can modulate ASD behaviors in mice [124,125,126]. Hsiao et al. found that GI barrier defects and microbiota alterations in the MIA offspring and oral treatment of MIA offspring with the human commensal *Bacteroides fragilis* improved their gut permeability and altered their microbial composition. Additionally, this treatment ameliorates defects in anxiety-like behavior, ultrasonic vocalization, the marble burying test with stereotyped behavior, and the pre-pulse inhibition (PPI) test with sensorimotor gating [125]. They also demonstrated that specific metabolites are altered in MIA offspring and normalized by *Bacteroides fragilis* treatment, with at least two molecules (4-ethylphenylsulfate and indole pyruvate) having potential relevance to ASD. In addition, reflecting another alteration in Trp metabolites, serum 5-HT was also increased. Sharon et al. demonstrated that transferring human ASD gut microbiota into germ-free mice is sufficient to induce hallmark ASD-like behaviors [126]. They also find that microbiota from ASD and typically developing (TD) individuals produce differential metabolome profiles in mice. Especially, 5-aminovaleric acid (5AV) and taurine, which are weak GABA_A_ receptor agonists, are significantly decreased in ASD offspring. Lower levels of GABA agonists show that gut microbes may impact inhibitory GABA signaling in the brain, which is related to ASD behaviors.

All accumulated evidence clarifies that the gut microbiota is either directly or indirectly associated with the pathogenesis of ASD. Microbial balance may influence brain development through the neuroendocrine, neuroimmune, and autonomic nervous systems. Additionally, the changes of Trp-related metabolites in the intestine may significantly impact the host’s physiological conditions and brain function.

## 4. Perinatal KP Metabolism

### 4.1. Neurodevelopment and KP Metabolites

**Figure 1:**
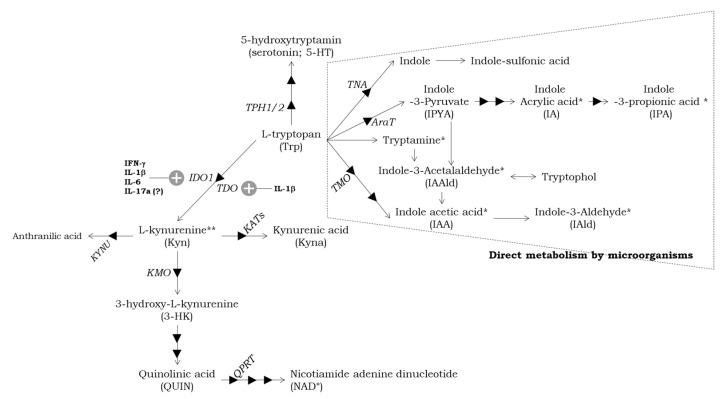
Simplified diagram of the Tryptophan (Trp) metabolism through serotonin (5-HT) and kynurenine (Kyn) pathways and direct metabolism by microorganisms. Pro-inflammatory cytokines, which upregulate enzyme activity of indoleamine 2,3-dioxygenase1 (IDO1) and tryptophan 2,3-dioxygenase (TDO), are highlighted by “+”. The triangle shows enzymes. Trapezoid with dotted line shows intestinal microbial pathway. TPH, tryptophan hydroxylase; KMO, kynurenine 3-monooxygenase; KATs, kynurenine aminotransferases; KYNU, kynureninase; QPRT, quinolinic phosphoribosyltransferase; TNA, tryptophanase; AraT, aromatic amino acid aminotransferase; TMO, tryptophan 2-monooxygenase. * Aryl hydrocarbon receptor (AhR) ligands, ** potential AhR ligand.

KP metabolite levels in the fetal brain are higher during the perinatal period [127], decrease in the immediate postnatal period, and remain lower in adulthood under physiological conditions [128]. A recent systematic review also indicated that physiological pregnancy requires a tight balance of KP metabolites [129]. A major source of these KP metabolites is provided from the mother to the fetus via transplacental transfer and Trp degradation in the placenta [128]. As mentioned in previous parts, the placenta expresses IDO1/TDO, and also other KP enzymes, such as kynureninase, kynurenine aminotransferase (KAT), kynurenine 3-monooxygenase (KMO), and quinolic acid phosphoribosyltransferase (QPRT) (Figure 1). In line with the expression of KP enzymes, its metabolites, Kyna, 3-hydrooxygense (3-HK), and quinolinic acid (QUIN), have been detected in the placenta [128]. Prenatal administration of Kyn and KMO inhibitors leads to biochemical and structural abnormalities in the rat hippocampus [130,131]. Previous studies showed that prolonged administration of a high concentration of Kyn to pregnant mice or rats results in elevated Kyn and Kyna (but not 3-HK) levels in specific areas of the fetal brain, parallel with distinct abnormal social behaviors and cognitive abnormalities in adult offspring [3,23,132]. Kyna, which is produced primarily by irreversible enzymatic transamination of Kyn, is an endogenous antagonist of the N-methyl-D-aspartate (NMDA) receptor [133] and α7 nicotinic acetylcholine receptor (α7nAChR) [134], and the levels of Kyna in the fetal brain under physiological conditions are high in several species, including mammalians [135]. Although Kyna does not cross the BBB in adulthood [136], it can directly transfer to the fetal or neonatal brain from circulation [137]. In the postnatal brain, Kyna levels immediately decline after birth. However, elevated Kyna levels have been found in the postmortem brain and cerebrospinal fluid of individuals with psychiatric disorders. One is SCZ, a severe mental disease from early neurodevelopment [11]. In experimental animals, increased brain Kyna concentrations during the perinatal period cause several cognitive impairments, consistent with that reported in SCZ patients [3,23,132]. Additionally, endogenous Kyn and Kyna levels are markedly increased in KMO-gene-deficient mice brains, and offspring of KMO-gene-deficient mice exhibit anxiety- and depression-like behavior [138] and several ASD-like behaviors [139]. Therefore, high Kyn, Kyna, or both levels in the brain may have a specific role in the normal and abnormal neurodevelopment of the fetus. However, little is still known about the roles of KP metabolites during the neurodevelopment process, and further investigation under physiological/pathological conditions is required to understand the ASD etiology.

### 4.2. Relation between Key Receptors and KP Metabolites in Developing Brain

During early brain development, NMDA, α7nACh, and aryl hydrocarbon receptors are key receptors targeted by KP metabolites. NMDA receptors are one of the glutamate receptors and are involved in neuronal cell migration [140], neurogenesis [141], axon guidance, synapse formation [142], and spine density [143]. Clinical studies on ASDs have identified genetic variants of NMDA receptor subunit genes. Specifically, de novo mutations have been identified in the *GRIN2B* gene, encoding the GluN2B subunit [144,145,146,147,148]. Additionally, many single nucleotide polymorphisms (SNPs) of *GRIN2A* (GluN2A subunit) and *GRIN2B* are linked with ASDs [149]. Pharmacological research shows that NMDA receptor agonist [D-cycloserine (DCS)] or antagonists (Memantine) can modulate ASD-related symptoms, including social deficits, stereotypy, and cognitive impairments [150,151,152,153]. Furthermore, animal studies have supported the contribution of NMDA receptor dysfunction to ASDs. In parallel with human research, positive or negative modulation of NMDA receptors can also normalize animal ASD-like behavior [154,155,156,157,158]. Generally, with clinical and animal studies, these results indicated that the optimal range of NMDA receptor function is important, and deviations in either direction can lead to shared behavioral impairments [159].

The homomeric α7nAChR subtype is abundantly present in the CNS/peripheral tissues and plays a key role in synaptic plasticity and various disease pathogenesis [160,161]. Several studies have shown a highly regulated expression of α7nAChRs in the developing brain during periods critical for establishing synaptic plasticity. A widespread distribution of α7nAChR mRNA is reported throughout the embryonic mouse nervous systems, highlighting the ubiquitous expression of α7nAChR mRNA in the central, peripheral, and enteric nervous systems during embryonic development [162]. In addition, the role of α7nAChRs in the pathogenesis of ASD has been investigated by several experimental and clinical studies. A larger and increased number of neurons were reported in the basal forebrain, a site of origin of cholinergic projections in the CNS, in children with ASD. In contrast, smaller and fewer neurons were reported in adults with ASD than in controls, indicating a functional disruption of cholinergic transmission in patients with ASD [163]. Pharmacological administration of a selective α7nAChR agonist (choline) from the beginning of pregnancy throughout lactation attenuated some of the deleterious ASD-like behaviors following MIA on the development of the offspring [164]. Additionally, the BTBR T+Itpr3tf/J mouse (BTBR), identified only a decade ago as displaying strong and consistent ASD-relevant behaviors, has shown decreased ACh levels and increased levels of Kyna in the medial prefrontal cortex [165]. Pharmacological administration of α7nAChR agonist (ALV-3288 or nicotine) significantly attenuated the deleterious ASD-like behaviors in BTBR mice [166,167]. Administering the acetylcholinesterase inhibitor or positive allosteric modulator of α7nAChR to children with ASD showed beneficial effects in clinical trials [168,169]. Moreover, it is well known that the mutations of the human chromosome 15q13.3 have been identified in the context of multiple neurological and psychiatric disorders, such as ASD and SCZ [170]. One of the striking genes in 15q13.3 is *CHRNA7*, which encodes α7nAChR [171]. In a clinical setting, significantly decreased levels of expression of *CHRNA7* have been revealed in the frontal cortex of patients with Rett syndrome, one of the neurodevelopment disorders strongly associated with ASD [172]. All these findings indicate that regulating α7nAChR activity during neuronal development is important, and endogenous Kyna may help balance the activation of these receptors.

Kyna, an astrocyte-derived product of the KP, is well-known for its neuroprotective and neuroinhibitory properties, which have been attributed to its action as a competitive antagonist at the glycine site on NMDA receptors with higher concentrations [173] and the allosteric site on the α7nAChRs at physiological levels [134]. Even though the sensitivity to inhibition of α7nAChR by Kyna is age-dependent [174], several electrophysiological and animal experiments show that α7nAChRs are the preferential target of endogenous Kyna in the brain during neurodevelopment [134,175,176]. α7nAChR is abundantly expressed in the CNS and located at pre- and postsynaptic sites [177], and neuronal α7nAChRs appear early during brain development [162]. Additionally, functional nAChRs responses can be found not only in neurons, but also in non-excitable cells, including microglia [178], astrocytes [179], Schwann cells [180], and other non-neuronal tissues [181], and these responses are often mediated by α7nAChRs. Notably, even relatively modest increased levels of Kyna in the brain negatively modulate the release of several neurotransmitters, such as glutamate [182,183,184], GABA [185], dopamine [186], and ACh [187]. These neurotransmitters are essential to the function of complex neural systems. Furthermore, there is evidence that α7nAChRs regulate the GABA_A_ receptor function and the developmental GABAergic switch from excitation to inhibition in ganglia and hippocampal neurons [188,189,190]. During critical phases of brain development, impaired neurotransmitter functions disrupt the maturation of the excitatory/inhibitory balance in cortical transmission, resulting in cognitive impairments and social abnormalities in ASD, SCZ, or both. Therefore, excessive blockage of α7nAChRs by abnormally elevated Kyna may be related to these behavioral deficits [5,11,176]. Further research will be needed to investigate the effect of Kyna on the maturation of the excitatory/inhibitory balance and other synaptic transmission systems during neuronal development.

AhR is a ligand-activated transcription factor that regulates cell differentiation, proliferation, and cancer imitation. Therefore, activation of AhR is related to the pathogenesis of several diseases, such as cancer, cardiovascular disease, inflammatory diseases, atherosclerosis, and neurodegenerative diseases [191]. In adults, AhR is widely distributed and expressed in various tissues and regions of the brain [192]. However, expression of AhR during fetal development is very limited in the placenta and epithelial cells of the fetus in physiological conditions. An experimental study shows that excessive activation of AhR signaling in neurons during embryonic development disrupts neuronal migration in the hippocampus [193]. It shows that AhR overactivation impairs neuronal growth and the neuronal circuit structure. In addition, during the developmental period, mice exposed to 2,3,7,8-tetrachlorodibenzo-p-dioxin (TCDD), which is the strong ligand for AhR, were shown to reveal AhR in neurons of the locus coeruleus (LC) and the island of Calleja major (ICjM) [194]. Kyn and Kyna are well-known to have AhR-ligand activity [62,195,196]. AhR-gene-deficient mice demonstrated increased levels of Kyna in specific brain areas associated with higher expression of KAT II. Furthermore, these animals were protected against neurological damage of excitotoxic QUIN by high levels of Kyna [197]. Generally, these studies disclose the multiplicity of biological actions of Kyna in the CNS, depending on the neuronal physiological condition.

### 4.3. Kynurenine Profile in ASD

There is increasing evidence that altered immune responses play a role in the pathogenesis of ASD, together with dysfunction of the glutamatergic and serotonergic systems. A few studies investigated the pro-inflammatory cytokine profile in patients with ASD [43,198]. In contrast, others focused on glutamatergic imbalance and toxicity as neuroinflammation markers [8] and dysfunction of the serotoninergic systems as diagnostic markers [199,200]. Among the KP metabolites, QUIN is involved in neurotoxicity during several inflammatory neuronal diseases because QUIN can activate NMDA receptors, increase neuronal activity, and elevate the intracellular calcium concentration [133,201]. Excessive activation of NMDA receptors leads to consequent impairment of the cytoskeleton homeostasis, with mitochondrial dysfunction and cell death induction [202]. High cerebral levels of QUIN, working as an NMDA agonist, can alter the excitation/inhibition ratio of the NMDA receptor and increase neuronal glutamate release, inhibiting its reuptake by astrocytes, and blocking astroglial glutamine synthetase, leading to excessive micro-environmental glutamate concentrations [203]. Lim et al. reported that an increased Kyn/Trp ratio and the production of Kyn and QUIN in children with ASD were consistent with increased levels of several pro-inflammatory cytokines, including IL-6 and IL-17a [8]. However, there were no significant changes in the concentration of Kyna compared to healthy children. However, other research shows no changes in Kyn and QUIN and lower levels of Kyna in ASD children [1].

Additionally, Carpita et al. investigated the changes in KP metabolites in ASD adults, and they found significantly lower levels of Trp and QUIN and no changes in Kyn and Kyna [2]. Recently, a meta-analysis comprising all KP metabolites and keywords related to maternal pregnancy and the fetal outcome showed that an altered KP metabolite concentration is significantly related to a high risk of preeclampsia, fetal growth restriction, and preterm birth [129]. These are all considered risk factors for ASD [204,205,206]. Even though single compound changes of KP are still controversial in each clinical study (Table 1) [1,2,8,207,208,209,210,211,212,213,214,215,216,217,218,219], it is clear that an abnormal balance of KP metabolites may be associated with adverse fetal neurodevelopment and the pathogenesis of ASD.

In addition to altered KP metabolites, it is well-known that ASD patients display high 5-HT blood levels [200,220,221] and decreased levels of central 5-HT [222]. The gene that encodes *TPH*, a rate-limiting enzyme for 5-HT synthesis, has been associated with ASD and other neurological disorders [223,224]. Moreover, a systematic review of common genetic variation in ASD showed that enriched pathways in the over-representation analysis are mostly associated with neurotransmitter receptors and their subunits, including 5-HT, GABA, and glutamate receptors [225]. During brain development, 5-HT has been shown to modulate numerous events, including cell division, neuronal migration, cell differentiation, and synaptogenesis, even before it becomes important as a neurotransmitter [226,227]. A pharmacological study showed that the administration of the 5-HT-depleting drug (*p*-chlorophenylalanine) to pregnant rats delays the onset of the differentiation of 5-HT neurons [228]. Consistently, human ASD model mice with 15q11.13 duplication (*15q dup* mice) show lower levels of 5-HT in all brain regions during developmental stages, and pharmacologically restoring 5-HT levels in *15q dup* offspring improves the cortical excitation/inhibition balance and rescues the impaired social behavior in adulthood [222]. Metabolized from Trp, 5-HT has been long since described as reducing 5-HT levels caused by Trp depletion [229,230]. Most dietary Trp (~95%) is metabolized via KP in the liver, and only a small fraction of the Trp pool is converted to 5-HT. In addition, a systematic review of the effects of acute Trp depletion on human brain function shows that acute Trp depletion impairs the consolidation of episodic memory for verbal information involved in ASD pathology [231]. Therefore, even small changes in the activity of the KP can significantly impact the Trp pool and normal levels of 5-HT in the brain during neuronal development.

## 5. Experimental Animal Models with Pre- and Postnatal KP Confusion to Study Neuropathological and Behavioral Deficits

As described in previous parts, the levels of KP metabolite in the developing brain are altered by environmental stimuli, including maternal infection and stress-causing MIA. It is well-known that maternal infections or stress are the most influential environmental risk factors for the offspring to develop psychiatric disorders, such as SCZ, depression, and ASD [6,232,233]. Furthermore, in experimental animal models, it is shown that MIA or maternal stress during pregnancy altered maternal and fetal KP metabolites [3,234,235]. Recent evidence has been accumulated on the impact of long-lasting changes of KP metabolites in the offspring involved in neuropathological and behavioral deficits (Table 2) [3,5,23,130,138,139,165,236,237,238,239,240,241,242,243,244,245,246,247,248,249,250,251,252].

Impaired KMO function has been implicated in the pathophysiology of SCZ [9,10,11,257] because decreased KMO activity is directly related to elevated levels of Kyna. Either pharmacological KMO inhibition or genomic deletion of the KMO gene in mice demonstrated reduced KMO activity and induced a shift in KP metabolism toward increasing the levels of Kyna. KMO-gene-deficit mice demonstrate increased basal Kyna from late gestation at E17-18 [128]. Conversely, 3-HK concentrations are almost undetectable in the placenta and fetal brain [138,258]. KMO-gene-deficient mice exhibited depressive-like behaviors, such as decreased sucrose preference and increased immobility in the forced swimming test, and the administration of antidepressants, selective serotonin reuptake inhibitors (SSRIs), can reverse these depressive-like behaviors [138]. In addition, these mice showed impairments in contextual memory, spent less time than controls interacting with an unfamiliar mouse in a social interaction paradigm, and showed increased anxiety-like behavior in the elevated plus maze and a light-dark box test. However, KMO-gene-deficit mice show an abnormally large increase in locomotor activity, compared to wild-type mice, when challenged with D-amphetamine and do not display disruption in PPI [139]. Pharmacological inhibition of KMO using 3,4-dimethoxy-N-[4-(3-nitrophenyl)thiazol-2-yl]benzenesulfonamide 16 (Ro 61-8048) during the pregnancy also resulted in distinct and long-lasting increased levels of Kyna, and significantly decreased expression of GluN2A and increased expression of GluN2B in the embryo brain [242]. The adolescent offspring at postnatal day (PND) 21 exhibited increased neuronal excitability, with increased levels of the Glu2A/Glu2B subunit of NMDA receptor and postsynaptic density protein (PDS)-95 [242,243]. Additionally, adult offspring (PND60) exhibited decreased overall numbers and lengths of hippocampal dendrites, together with fewer dendritic spines and less dendritic complexity, and disruptions in long-term potentiation (LTP) [130,131]. The number of neuron terminals staining for vesicular glutamate transporter (VGLUT)-1 and VGLUT-2 was significantly increased by Ro 61-8048 treatment, with no changes in the expression of vesicular GABA transporter, showing that prenatal inhibition of the KP produces marked effects on neuronal structure and the excitatory/inhibitory balance.

A different approach is directly increasing Kyn levels during pre- and postnatal development by feeding Kyn-laced chow or administrating Kyn to the pregnant dam continuously. Pocivavsek et al. demonstrated that continuous feeding of Kyn-laced chow from E15 to 22 elevated the levels of Kyna in the rat brain during the entire treatment period. Biochemical and behavioral tests in adulthood showed distinct changes, such as decreased expression levels of GluN2A and a trend toward decreased α7nAChR expression, and lower performance in trace fear conditioning tests [245]. The adult offspring also displayed behavioral impairments in hippocampus-related cognitive tasks, such as a lower passive avoidance perform and the Morris water maze [23]. Embryonic Kyn-exposure male offspring also displayed reduced rapid eye movement (REM) sleep, indicating prenatal Kyn elevation impairs sleeping behavior in rats [246], and ASD people are often reported to have sleeping troubles. In addition, they found time-of-day- and sex-dependent alterations in the levels of Kyna, glutamate, and GABA in the hippocampus, indicating that these hippocampal neuromodulations may be related to regulating memory consolidation, retrieval, and locomotor activity [247]. As a follow-up to these studies, continuous maternal Kyn administration in pregnant mice from E12.5 to 19 resulted in behavioral abnormalities, including social and cognitive defects. Kyn-injected adult offspring exhibited higher Kyn and Kyna in the fetal brain and lower performance on social recognition tests and novel object recognition tasks [3].

Additionally, neonatal Kyn-administered mice (PND7-16) showed an enhanced sensitivity to a D-amphetamine-induced increase in locomotor activity, mild impairment in PPI, and lower performance on the trace fear conditioning test, as shown in KMO-gene-deficient mice [251]. Furthermore, neonatal rats (PND7-10) administered Kyn exhibited decreased social interaction and locomotor activity, with long-lasting high concentrations of Kyna and QUIN in the brain [250]. Systemic administration of a high dose of Kyn in adult mice also disrupts their object recognition memory and decreases their locomotor activity [132]. Even a low dose of systemic Kyn injections in adult mice induces depression-like behavior [259].

However, KAT-II-gene-deficient mice show lower Kyna levels in the hippocampus and striatum and higher spontaneous locomotor activity, compared to wild-type mice during the early postnatal time-point (PND14 and 21), but no changes at PND60 [253,254]. At this age, KAT-II-gene-deficient mice exhibited a significantly increased performance in object exploration and recognition tasks, the passive avoidance test, and the spatial discrimination test, reflecting partly on the hippocampal function. Additionally, hippocampal slices from KAT-II-deficient mice showed a significant increase in the amplitude of LTP in vitro compared to wild-type controls [183]. Moreover, the α7nAChR activity induced by exogenous application of agonists to hippocampal stratum radiatum interneurons was extremely higher in KAT-II-gene-deficient mice than that in wild-type mice [253]. Pharmacological administration of selective KAT II inhibitor raised extracellular dopamine levels in the striatum [260], inhibited the firing rate and burst activity of dopamine neurons in the midbrain area, and reduced the number of spontaneously active dopamine cells. Pretreatment with an agonist of the NMDA receptor prevented the inhibitory action, and pretreatment with an antagonist of the GABA_A_ receptor partially prevented the inhibitory effect on the KAT II inhibitor’s firing rate and burst firing activity. Therefore, the effect of KAT II inhibitor appears to be specifically executed by NMDA receptors and mediated indirectly via GABA_B_-receptor-induced disinhibition of dopamine neurons [261]. However, intrastriatal infusions of neurotoxic QUIN resulted in dose-dependent lesions, and the striatal damages is larger in KAT-II-gene-deficient mice than in wildtype mice in PND14 when the Kyna levels are lower, but no difference in the lesion volume at PND60 has been reported [254].

Generally, all studies show that time- and dose-dependent optimal levels of KP metabolites during critical neurodevelopmental periods are essential for normal brain development. Additionally, an imbalance of KP metabolites induces long-lasting changes relevant to various psychiatric diseases.

## 6. Conclusions

The immune system plays an important role in neurodevelopment and multiple neurobiological functions. Exposure to maternal immune activation during early pregnancy has been identified as the most influential environmental risk factor for ASD. Recent evidence shows that a responsible inflammatory pathway in MIA-associated ASD is related to the activity of Th17 lymphocytes and their effector, cytokine IL-17a, among immunological factors. The association of IL-17a in the etiology of ASD has been found not only in human clinical studies of patients with ASD, but also in MIA model experimental research. Therefore, there is no doubt that IL-17a dysregulation may play a causal role in the development of ASD. However, it is still controversial whether IL-17a can pass through the placental barrier and directly affect fetal brain development.

**Figure 2:**
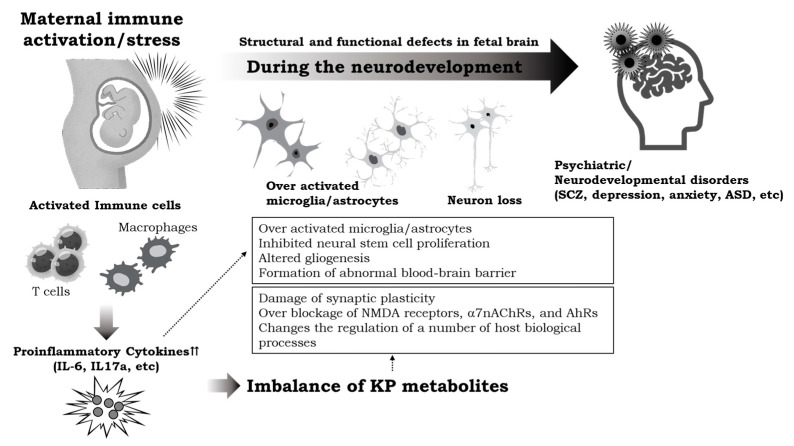
Summary of the review.

However, it is clearly shown that abnormal levels of KP metabolites, especially Kyn and Kyna, during neurodevelopment have been related to several neurobiochemical and behavioral impairments associated with depression, anxiety, ASD, and SCZ phenotypes (Figure 2). During early pre- or postnatal fetal development, activation of the KP, with an increase in neurotoxic metabolites and excitotoxicity, causing long-term changes in glutamatergic/GABAergic functions, trophic support, and synaptic function, may be linked to various psychiatric disorders. Yet, to validate the direct connection between neuroinflammation and KP metabolites in the pathology of ASD, some information is missing, and wide translational research is necessary. All recent research on the imbalance of KP metabolites during brain development show that interventions aimed at directly reducing KP metabolites or their sites of actions at critical periods may shed light on novel therapeutic strategies not only for neurodevelopmental disorders, but also to prevent the manifestation of neuropsychiatric and other CNS disorders.

## Figures and Tables

**Table 1:** Changes of kynurenine (Kyn) metabolites’ profile in ASD patients.

Age (Mean ± SD, Range)	Country	Specimen	Diagnostic Criteria	Detection Method	Observation	Ref.
11.2 ± 2.02 y	Norway	Serum	ADI-R, ADOS, ICD-10	HPLC	Kyna (⇈), QUIN (⇈)	[1] [207]
8.47 ± 2.36 y, 3-10 y	Australia		DSM-IV, CARS	UHPLC	Kyn (⇈), QUIN (⇈)	[8]
35.5 ± 9.9 m	Turkey		DSM-5, CARS	HPLC	3-HK (⇈), Kyna (⇈)	[208]
42.86 ± 11.03 m	China		DSM-IV, ADI-R, ADOS	TMS/MS	Trp (⇊)	[209]
3.46 ±0.56 y, 2–6 y	China	Plasma	DSM-IV	LC-MS/MS	Trp (⇊)	[210]
4.4 ± 1.7 y	India		DSM-IV, ABC	Reverse-phase HPLC	Trp (⇊)	[211]
3.22 ± 1.18 y, 1–14 y	China		DSM-5, ADI-R, ADOS	LC-MS/MS	Trp (⇊)	[212]
10.0 ± 3.1 y, 5–16 y	USA		DSM-IV	LC-MS/MS	Trp (⇊)	[213]
27.75 ± 6.97 y	Italy		DSM-5,RAADS-14,RRS, WSAS	ELISA	Trp (⇊), Kyna (⇊)	[2]
4–16 y	Italy	Urine	ADOS	GC-MS	Trp (⇈)	[214]
4–10 y	Poland		DSM-IV	GC-MS	Trp (⇊)	[215]
4.83 ± 2.40 y, 3–7 y	Italy		DSM-IV, ADI-R, ADOS, CARS	HILIC-UHPLC	Kyn (⇊), Kyna (⇊), QUIN (⇈), 5-HT (⇊)	[216]
7.06 ± 0.96 y	Italy		DSM-IV, DSM-5, ADI-R, ADOS	UHPLC	Kyn (⇊)	[217]
3–6 y	Armenia		ADI-R, ADOS, DSM-IV	LC-MS/MS	QUIN (⇈)	[218]
6.1 y, 33 m-10 y	USA	CSF	DSM-IV, ADI-R	GC-MS	QUIN (⇊)	[219]

Significantly decreased (⇊), significantly increased (⇈). y, years old; m, months old; ADI-R, Autism Diagnostic Interview-Revised; ADOS, Autism Diagnostic Observation Schedule; ICD-10, International Statistical Classification of Diseases and Related Health Problems-10; HPLC, high-performance liquid chromatography; DSM-IV, Diagnostic and Statistical Manual of Mental Disorder, Fourth Edition; CARS, Childhood Autism Rating Scale; UHPLC, ultra-high-performance liquid chromatography; DSM-5, Diagnostic and Statistical Manual of Mental Disorder, Fifth Edition; TMS/MS, tandem mass spectrometry; LC-MS/MS, liquid chromatography—tandem mass spectrometry; ABC, Aberrant Behavior Checklist; RAADS-14, Ritvo Autism & Asperger Diagnostic Scale-14; RRS, Ruminative Response Scale; WSAS, the Work and Social Adjustment Scale; ELISA, enzyme-linked immunosorbent assay; GC-MS, gas chromatography—mass spectrometry; HILIC, hydrophilic interaction chromatography.

**Table 2:** Effect of disrupted Kynurenine (Kyn) metabolites during the neurodevelopmental period in animal models.

Study Model	Species	Changes of Kyn Metabolites in Offspring	Behavioral Changes	Effect on Brain	Ref.
Poly(I:C) i.p. at E9.0–9.5	Mice	Trp (⇊), Kyn (⇊), QUIN (⇈), KMO (⇈), 5-HT (⇊), QUIN/Kyna ratio (⇈) after PND80	Impaired working memory Increased anxiety-like behavior Impaired attentional set shifting after PND80	5-HT_2A_ (⇈), SERT (⇈), m*Glu2* (⇊) Functional GABAergic transmission (⇊) Decreased PV^+^ interneuron transmission after PND80	[236] [237] [238]
Poly(I:C) i.p. at E14, 16, 18	Rat	Kyn (↑) 5 h after injection		GluN2A (↓), GluN2B (⇈), DCX (⇊) 5 h after injection	[239]
Poly(I:C) i.p. at E9.0–9.5	Mice	Trp (⇊), Kyn (⇊), QUIN (⇈), KMO (⇈), 5-HT (⇊), QUIN/Kyna ratio (⇈) after PND80	Impaired working memory Increased anxiety-like behavior Impaired attentional set shifting after PND80	5-HT_2A_ (⇈), SERT (⇈), m*Glu2* (⇊) Functional GABAergic transmission (⇊) Decreased PV^+^ interneuron transmission after PND80	[240]
KMO inhibitor i.p. at E14, 16,18	Mice	Kyn (⇈), Kyna (⇈) 5 and 24 h after injection			[241]
KMO inhibitor i.p. at E14, 16, 18	Rat	Kyn (⇈), Kyna (⇈) 5 and 24 h after injection	Does not affect the initial acquisition nor the subsequent memory consolidation at PND60	GluN2A (⇈), GluN2B (⇈), PSD-95 (⇈) Increased LTP at PND21 GluN2A (⇊), GluN2B (↓), PSD-95 (↓), VGLUT-1(⇈), VGLUT-2 (⇈), Decreased LTP and basal spine densities in CA1 at PND60	[130] [242] [243]
IL-17a high expression at E12.5-PND0 Kyn i.p. at E12.5–19	Mice	Kyn (⇈), Kyna (⇈) Kyn/Trp ratio (⇈) at E18.5 TRP (⇊), Kyn/Trp ratio (⇈) at PND77	Impaired cognitive function Lower social behavior and social cognition Depressive-like behavior at PND 56-70		[3]
Kyn i.p. at E14,16,18	Rat			GluN1 (↑), GluN2A (↑), GluN2B (↑) Decreased LPT	[244]
Fed high Kyn diet at E15–22	Rat	Kyn (⇈), Kyna (⇈) at E22 Kyna (⇈) at PND56	Impaired spatial learning, reference memory, and contextual memory Impaired attentional set-shifting at PND 56-85 Reduced rapid eye movement sleep at PND85	m*GluR*2 (⇊) at E21 m*Chrna*7 (⇊) at PND2 Decreased dendritic spine density at PND56-80 m*Grin1* (⇊), m*Grin2a* (⇊) at PND70 Time- and sex-dependent alterations in the levels of GABA and glutamate at PND56	[5] [23] [245] [246] [247]
Fed high Kyn diet at E15–PND21	Rat	Kyn (⇈), Kyna (⇈), 3-HK (⇈) at PND21	Impaired spatial learning, reference memory, and contextual memory Impaired attentional set-shifting at PND56	Decreased extracellular glutamate	[248] [249]
Kyn i.p. at PND7–10	Rat	Kyna (⇈), QUIN (⇈) at PND10 Kyna (↑), QUIN (↑) at PND70	Impaired social behavior Decreased locomotor activity		[250]
Kyn i.p. at PND7–16	Mice	Kyna (⇈) at PND16	Decreased amphetamine-induced locomotor activity Impaired PPI, working memory	Enhanced amphetamine-induced dopamine release	[251]
KMO gene- deficit	Mice	Kyn (⇈), Kyna (⇈)	Impaired contextual memory Decreased social interaction Increased anxiety- and depressive-like behavior Increased amphetamine-induced locomotor activity	Increased spontaneous ventral tegmental area dopamine neuron activity Decreased LPT	[138] [139] [244] [252]
KAT II gene-deficit	Mice	Kyna (⇊), QUIN(↓) at PND14 and 21 No significant changes at PND60	Increased performance in object exploration and recognition tasks, passive avoidance test, spatial discrimination test, and spontaneous locomotor activity at PND21	Increased LPT Increased extracellular glutamate concentrations and endogenous α7nAChR activity	[183] [253] [254]
BTBR T+Itpr3tf/J	Mice	Kyna (⇈)	Display autism-relevant behaviors	Absence of the corpus Severely reduced hippocampal commissure	[165] [255]
Mecp2^+/−^	Mice	No significant changes	Exhibit hindlimb clasping and uneven breathing Model of human Rett Syndrome	Reduced in global and all local volumes in the brain	[241] [256]

Significantly decreased (⇊), significantly increased(⇈), decreased (↓), increased (↑) but not statically significant. SERT, serotonin transporter; PV^+^, parvalbumin positive; DCX, doublecortin.

## Data Availability

Not applicable.

## Abbreviations

ABC	Aberrant Behavior Checklist
ADDM	Autism Developmental Disabilities Monitoring
ADI-R	Autism Diagnostic Interview-Revised
ADOS	Autism Diagnostic Observation Schedule
AhR	aryl hydrocarbon receptor
APCs	antigen-presenting cells
ASD	autism spectrum disorder
5AV	5-aminovaleric acid
Breg	B-regulatory cell
BBB	blood-brain barrier
CARS	Childhood Autism Rating Scale
CNS	central nervous system
DCS	D-cycloserine
DCX	doublecortin
DSM	Diagnostic and Statistical Manual of Mental Disorder
E	embryonic day
ELISA	enzyme-linked immune sorbent assay
*E. coli*	*Escherichia coli*
*15q dup*	15q11.13 duplication
GABA	gamma-aminobutyric acid
GS-MS	gas chromatography-mass spectrometry
GI	gastrointestinal
HDAC	histone deacetylase
HILIC	hydrophilic interaction chromatography
3-HK	3-hydroxy-L-kynurenine
HPLC	high performance liquid chromatography
5-HT	5-hydroxytryptamine
IA	indole acrylic acid
IAA	indole-3-acetic acid
IAAld	indole-3acetaaldehyde
IAld	indole-3-aldehyde
IBD	inflammatory bowel disease
ICD-10	International Statistical Classification of Diseases and Related Health Problems-10
ICjM	island of Calleja major
IDO1	indoleamine 2,3-dioxygenase1
IFN-γ	interferon-gamma
IL-1β	interleukin-1 beta
IL-6	interleukin-6
IL-17	interleukin-17
IL-21	interlekin-21
ILC3	innate lymphocyte cells 3
IPA	indole-3-propionic acid
KAT	kynurenine aminotransferase
KMO	kynurenine 3-monooxygenase
KP	kynurenine pathway
Kyn	kynurenine
Kyna	kynurenic acid
LC	locus coeruleus
LC-MS/MS	liquid chromatography-tandem mass spectrometry
LPS	lipopolysaccharide
LRP	low-density receptor-related protein
LTP	long-term potentiation
MIA	maternal immune activation
nAChR	nicotinic acetylcholine receptor
NMDA	*N*-methyl-D-aspartate
PDS-95	postsynaptic density protein-95
PND	postnatal day
poly(I:C)	polyinosinic:polycytidylic acid
PPI	pre-pulse inhibition
PV^+^	parvalbumin positive
QPRT	quinolinic phosphoribosyltransferase
QUIN	quinolinic acid
RAADS-14	Ritovo Autism & Asperger Diagnostic Scale-14
REM	rapid eye movement
Ro 61-8048	3,4-dimethoxy-N-[4-(3-nitrophenyl)thiazol-2-yl]benzenesulfonamide 16
RRS	Ruminative Response Scale
SCFA	short-chain fatty acid
SCZ	schizophrenia
SERT	serotonin transporter
SFB	segmented filamentous bacteria
SHP	SC-43 activated SH2 domain-containing phosphatase
SNP	single nucleotide polymorphism
SSRIs	selective serotonin reuptake inhibitors
STAT	signal transducers and activators of transcription
TCDD	2,3,7,8-tetrachlorodibenzo-p-dioxin
TD	typically developing
TDO	tryptophan 2,3-dioxygenase
TGF-®	transforming growth factor-beta
Th	T helper
TMS/MS	tandem mass spectrometry
TNF	tumor necrosis factor
TPH	tryptophan hydroxylase
Treg	T-regulatory cell
Trp	tryptophan
UHPLC	ultra-high performance liquid chromatography
VGLUT	vesicular glutamate transporter
WSAS	Work and Social Adjustment Scale
